# An online 11 kv distribution system insulator defect detection approach with modified YOLOv11 and mobileNetV3

**DOI:** 10.1038/s41598-025-99756-5

**Published:** 2025-05-05

**Authors:** Arnav Bhagwat, Soham Dutta, Debdeep Saha, Maddikara Jaya Bharata Reddy

**Affiliations:** 1 Department of Computer Science, University of Exeter,, Exeter, UK; 2 Department of Electrical and Electronics Engineering, Manipal Institute of Technology, Manipal Academy of Higher Education, Manipal, 576104 Karnataka India; 3 Grid Edge Solutions, IDC, Hitachi Energy Services Pvt. Limited, Bengaluru, India; 4 Department of Electrical and Electronics Engineering, National Institute of Technology, Tiruchirappalli, 620015 Tamil Nadu India

**Keywords:** YOLO V11, Unmanned aerial vehicles, Distribution automation system, Insulator defect detection, MobileNetV3, Electrical and electronic engineering, Mechanical engineering

## Abstract

With the advent of smart distribution grids, detection of defects in insulators with unmanned aerial vehicles as a part of distribution automation system (DAS) has attained a widespread attention. The defects are essential to detect to avoid damaging the service life of distribution lines, serious power loss and cascading power outages in extreme conditions. The intricate background, limited image dataset and small-scale object makes the problem of detection more complex. Owing to the exponential advancement in deep learning, deep learning-based insulator defect detection is gradually attaining a foothold in the research domain. This paper presents a novel approach for detecting insulator defects in an 11 kV distribution system using a modified version of You Only Look Once (YOLO V11) and the MobileNetV3 model. Data augmentation was applied as part of the preprocessing phase to train the proposed model. The model’s performance was compared with earlier versions of YOLO and other existing methods to demonstrate its effectiveness. Additionally, multiple case studies were conducted to validate the method’s robustness and reliability for insulator defect detection. This paper incorporates a modified version of YOLOv11 architecture using the constituent C3K2, SPFF and C2PSA algorithmic blocks, mounted with a MobileNetV3 classifier to allow lightweight framework in DAS based devices. Studies involving various real-life scenarios show the efficacy and applicability of the proposed algorithmic pipeline.

## Introduction

As a vital part of power scrutiny, insulators have a major role in ensuring a reliable power supply in distribution lines. These distribution lines are constantly exposed to variation of climate, dust and chemicals. These leads to the increase in conductivity of the insulation surface making it more susceptible to discharge. Moreover, due to aging or some external shock, the ceramic or glass material of the insulators can crack. The insulators lose their property if their surface gets charred. In all these cases, the insulators must be replaced. Thus, the regular inspection of distribution insulators is essential due to the following reasons^1^:

i. It is a mandate for power distributors.
ii. It leads to reduced power outages if detected early.
iii. It ensures public safety.
iv. It helps the maintenance crew to be briefed accurately so that they are prepared for maintenance and repair job.

The classical approach of inspection was dependent on manual patrol, which was laborious, costly as well as dangerous. With the advent of drone technology, manual patrolling is gradually replaced by unmanned aerial vehicles (UAV). However, due to the increase in geographical sprawl of modern distribution system, distribution lines now pass through different landforms such as forests, lakes, deserts, etc. This leads to a complex background for UAV images. These complexities are further enhanced due to the increased number of high-rise buildings. Thus, the UAV images of the insulators suffer from inherent disadvantages such as blurring, occlusion and interference of other objects^2^. This makes it difficult to analyse the UAV images manually.

### Impetus of the research

The penetration of information technology in power sector has led to automation in various areas. It has enabled power system engineers to remotely take control actions. Distribution automation system (DAS) has emerged through system-wide miniaturization and incorporation of information technology^3^. As per IEEE, DAS system allows a power utility company to monitor, operate and coordinate various distribution components remotely in real time. The advantages of such a system are reduction in commercial and technical loss, reduction in damage of the equipment, better reliability and power quality, improved cash flow, high speed restoration of electricity, strategic operational planning and enhanced system information availability. Insulator defect detection is an integral part of DAS because insulator defects cause disruption to proper power flow. Thus, constant monitoring of the insulators is inevitable in a DAS environment. However, the task of automatic defect detection requires a robust and fast method capable of handling complex scenarios.

In the last ten years, insulator defect detection with computer vision methods has acquired its foothold. Classical computer vision-based techniques aim at conceiving a thumb rule to segregate defects and insulators from the background^4,5^. Precisely, it considers the texture, shape, colour and contours as vital facts for the process of detection. Nevertheless, these methods demonstrate inadequate robustness as insulators and their faults depict abundant variations along with their complex backgrounds. Even though these methods have achieved many improvisations, they still possess low reliability and are unable to meet the capability of accurately detecting insulator defects in the power line inspection business. Deep learning emerged in the early 2010 s. Since then, numerous studies have been conducted for insulator defect detection employing deep learning. In comparison with traditional methods, deep learning-based approaches exhibit more flexibility and adaptability in detecting insulator defect errands under different backgrounds. The you only look once (YOLO) versions are a target detection algorithm built up on the concept of deep learning and convolutional neural network. It possesses the advantages of high detection accuracy, high computational speed, and real-time monitoring^6,7^. Of all the versions, YOLOv11 is the latest version and displays excellent performance in defect detection. Owing to the above discussions, YOLOv11 has the possibility to emerge as a potential tool for insulator detection. MobileNetV3 is a neural network architecture conceived for mobile and edge devices^8^. It is developed by Google and is suitable for real-time image classification. Thus, it has the potential to be used for faulty insulator detection.

### Previous literature on insulator defect detection

There has been a plethora of research available in the literature in the field of insulator defect detection. During the pre-smart grid era, the defect detection was manually carried out by the patrolling team. However, with the advent of the smart grid era, the image processing method was employed for insulator defect detection. Some of these methods are based on local features and spatial orders^8^, spatial morphological features^4,9^, etc. These traditional image processing methods detected insulators with the aid of specific features such as texture, gradient, colour, shape, etc. before the implementation of fault detection by respective techniques. These methods suffer from the disadvantage of non-suitability for multi-scale detection in complex backgrounds as these methods rely on the classification features developed manually. Since the insulator images in practical scenarios have uncertain correlation features, the accuracy of these methods is not satisfactory.

Due to the exponential progress in deep learning concept, research has been inclined to deep learning with image processing as the backbone for detecting faults in insulators. These methods overcome the shortcomings of image processing-based methods for insulator faults^10–12^. The most commonly used deep learning model is convolutional neural network (CNN) which possesses the ability to extract features from images in an automated fashion and to learn from various environmental scenarios. One such work has been done in^13^where a Box-Point Detector converts the fault analysis to a combined job of box location and point estimation, which comprises of a CNN followed by two parallel branches of convolutional heads. The method has a quantitative performance of 94% accuracy on the testing data. Hence, deep learned methods exhibit a higher performance and higher capability of object detection when compared with image processing methods^14^. The available literature on deep learning-based insulator fault detection can be grouped into two groups – one-stage network and two stage networks^14^. Two-stage networks are regional recommendation approaches that have a region concerning the topic of interest as well as a separate region concerning classifying the item, initially by creating candidate regions of interest and finally executing the process of classification of extracted features. Some of the commonly used two-stage networks for insulator fault detection are regions with convolutional neural networks (R-CNN)^10^and their variations such as faster R-CNN^15^, etc. The two stage networks have the advantage of higher accuracy than one stage network but at the cost of increased computational time. To mitigate this issue, one stage network algorithms called Single Shot multi box Detector (SSD) and YOLO algorithms came into existence in the literature of insulator fault detection. A new technique based on multi-level perception by an SSD algorithm for the automatic detection of insulator faults is proposed in^16^. The method has a quantitative performance of 91.23% precision and 93.69% recall under a diverse background. Similar work has been done in^17^ which employed a deep learning model based on SSD. Experimental results show that the faults can be distinguished accurately and quickly by the presented monitoring algorithm.

Though the fault recognition pace of the SSD technique has been significantly enhanced in comparison to the two-stage networks, YOLO models exhibit a better running time^18^. In^19^, a real-time detection model based on YOLOv2 is presented for the automatic detection of insulators. It is experimentally shown that the technique can locate insulator on real-time UAV based image data accurately. After the detection, the images are further exposed to insulator surface condition assessment for the presence of water, snow and ice using various classifiers. A similar work has been done with YOLOv3 in^20^. An improved YOLOv3 network is also presented in^20^for insulator defect detection, where the feature pyramid networks of improved YOLOv3 is transformed into bidirectional fusion to improve the detection accuracy of small targets. Similar work has been done with YOLOv4 in^21^. In the same paper, four types of YOLOv5 series (YOLOv5 m, YOLOv5 s, YOLOv5 l and YOLOv5x) are presented to solve the problem of low insulator defect detection accuracy in UAV detection. The experimental results demonstrate that YOLOv5 have notably higher accuracy than earlier versions of YOLO. It is noted that for the YOLOv5 version, an increase in the intricacy of the model leads to a decrease in performance due to insufficient volume of data. The MTI-YOLO.

^22^technique presents multi-scale feature fusion structure, multi-scale feature recognition probe and spatial pyramid pool model for optimizing the running time, accuracy of detection and storing space. The method exhibited greater accuracy in complex cases, better memory consumption and running time in comparison to previous versions of YOLO. An optimal YOLO model and CNN structure is combined as Hybrid-YOLO in^23^for insulator fault detection for obtaining best results. The uncertainty problem of insulator detection is resolved in^24^ by application of a Gaussian with YOLO to obtain you only look only drone (YOLOD) model. The technique improves the boundary box forecast and refines the robustness of insulator fault detection. Previous implementations utilize YOLOv3 to YOLOv8 when it came to object detection and localization. Recent developments with model versions such as YOLOv7 and YOLOv8 show remarkable improvements in speed and detection accuracy. However, these models are significantly wider and deeper, making them less suitable for deployment on DAS environments and devices. EfficientNet and ElasticNet perform well but suffer from the problems of high latency and are not suitable for real time UAV image analysis. YOLOv11 showed an elegant balance between size, efficiency, speed and accuracy.

The authors in^25^proposed a novel deep-learning technique of MobileNetv3 to classify the face emotions in thermal images. The method is analyzed with two different thermal face expression databases - the IR database and the IRIS Thermal/Visible database. The method exhibited a better precision, accuracy, F1-score, and recall parameters when compared with other techniques of face detection. Similarly, an efficient and simple technique to improvise identification of grape leaf disease with limited computing resources and scale of training image dataset based improved MobileNetV3 model and deep transfer learning is presented in^26^. An improved Mobilenetv3-Yolov5 infrared target detection algorithm based on attention distillation is developed in^27^. The testing results on Ambarella platform depicted the models’ greater accuracy and running speed as compared to Yolov5, Yolov3, Yolov2 and FasterR-CNN with 91.9 mean average precision (mAP). The MobileNetV3 with Aquila optimizer is used for Covid detection in^28^. MobileNetV3 is employed as a backbone feature extraction. Aquila optimizer is employed for feature selection to lower the image and improve the accuracy of classification. Two datasets with X-ray and CT COVID-19 images are tested with the method. The algorithm realizes accuracy superior to other approaches in terms of performance metrics. YOLOv9, v10, and v11 algorithms are employed in^29^to detect defects in solar panels. YOLOv11 gave the best results. Similarly, damage detection in concrete structures with multi-feature backgrounds using the YOLOv3-v10 is proposed in^30^.

### Features of the work

The features of the work can be summarised in the following points:

1. A DAS architecture for automatic insulator defect detection is proposed which saves cost and time of the distribution operators. Smart remote terminal units are employed which has the capability of image processing and identify faulty insulators with machine learned algorithms.
2. The modified form of the latest version of YOLO i.e. YOLOv11 is used as the deep neural network algorithm for the insulator detection. The advantages of the modified version of YOLO are clearly depicted in the evaluation metrices when compared to the earlier versions of YOLO.
3. The method exploits MobileNetV3 as an ideal lightweight classifier for classification of insulators as healthy or faulty.
4. Practical data of a 11 kV electricity distribution network located inside the campus of National Institute of Technology, Trichy, India is taken for the development, validation and testing of the proposed algorithm.
5. The algorithm has high detection accuracy. Several test cases show the robustness of the algorithm.
6. The proposed algorithm is compared with other deep learned models for insulator fault detection. The evaluation metrics show the improved performance of proposed method over other methods.

### Organization of the work

The rest of the work is presented as follows. The “DAS Architecture for 11 kV Distribution Line Insulators Defect Detection” section deals with the defect detection architecture for 11 kV distribution line insulators where the process of implementing the algorithm in the distributed automation system environment is explained. Additionally, the information about the dataset used to validate the algorithm is stated in this section. In the “Methodology” section, the methodology of the algorithm is explained in detail. A brief theory about YOLOv11 and MobileNetV3 is explained along with each step of the development of the algorithm. The proposed algorithm is extensively tested for different test scenarios in the “Results and Discussion” section. The algorithm has been compared with other methods of classification as well as other insulator fault location techniques available in the literature. In the “Conclusion” section, inferences about the algorithm and the future works have been discussed.

## DAS architecture for 11 kv distribution line insulators defect detection

**Fig. 1:**
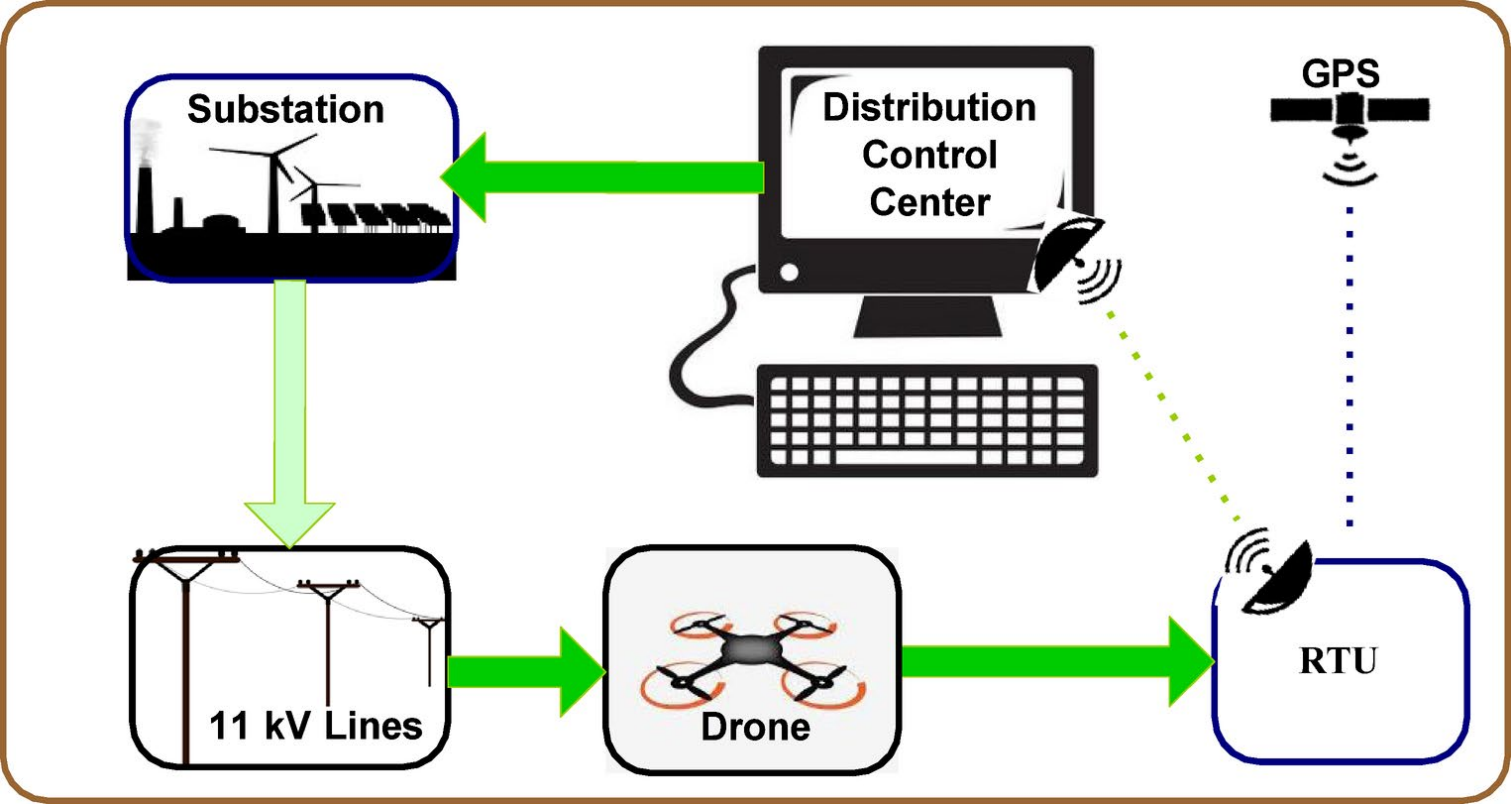
DAS architecture for 11 kV distribution line insulators defect detection.

The DAS architecture of the defect detection of 11 kV distribution line insulators is depicted in Fig. 1. The UAV acquires the images of the insulators periodically. Since the images are acquired frequently, short term weather effects such as cloud or fog do not affect the detection process. The acquired images are transferred to the smart remote terminal units where the images are processed by the methodology explained in the next section. If there is a defect detection by the remote terminal units, then the location of the insulators is sent to the distribution control center with the help of global positioning system. The control center takes appropriate decisions as per the health of the insulator. If the health of the insulator is poor, then a notification is sent to the local substation of the concerned deteriorated insulators regarding its replacement. Thus, the defect is located automatically without any intervention of humans.

**Fig. 2:**
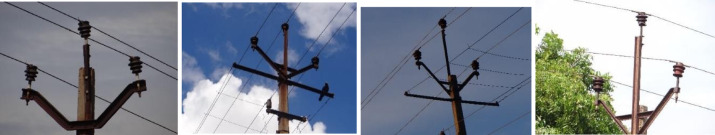
Some examples of captured images of 11 kV distribution line insulators.

The dataset considered in this study consists of UAV images of porcelain insulators mounted on cement poles of a 11 kV electricity distribution network located inside the campus of National Institute of Technology, Trichy, India. Some images are provided in Fig. 2.

## Methodology

### Brief theory on YOLOv11 for object detection

The YOLO algorithm is a convolution neural network that divides a particular sample image into a grid structure, with the model making predictions on each grid. YOLO is a highly efficient single shot detector. The YOLOv11 model involves the use of three highly innovative blocks to efficiently parse spatial information, namely, the C3 K2 block, the SPFF module, and the C2PSA block. It uses the convolution followed by normalization backbone common to most YOLO models, building additional blocks on this backbone^31^.

A. C3 K2 block: Allows the model to efficiently handle feature extraction. It works by splitting up the fed information into 3 × 3 sub-kernels. By splitting up information into these smaller convolutional chunks, it reduces the number of features that must be dealt with, thereby reducing computational complexity. The 3 × 3 kernels split the input image into smaller chunks while utilizing depth wise separable convolutions. This block has the primary function of vastly improving speed by reducing the trainable parameters, all while preserving accuracy. A reduction in model size of nearly 20% is seen over previous versions.
B. Spatial Pyramid Pooling Fast (SPFF) block: It retains the older SPFF block found in v8 versions, it allows the model to pool layer features from different parts of an image at different rates. This allows YOLOv11 to detect and differentiate between parts of images, especially small object detection, which did not always work with earlier yolo versions. The primary function of the proposed SPFF block is to efficiently capture both the global and local spatial patterns by using feature aggregation at the receptive fields. This block allows us to effectively identify small defects in the insulator which might not have otherwise been captured due to occlusion.
C. Cross Stage Partial with Spatial Attention block (C2PSA): This block employes the use of two partial sparse attention blocks, that refines the ability of the YOLO model to focus on selected regions of interest by applying spatial attention. This block allows the model to detect fine details in an image. The primary function of this module is to apply attention to relevant regions. This additional focus allows the model to focus on partial residual connections that prevent any kind of parameter explosion while still improving localization precision.

### Brief theory on mobileNetV3 for classification

MobileNetV3 emphasizes very quick and efficient deployment on mobile and embedded devices. Hence, it is an ideal lightweight classifier. MobileNetV3 performs much better than most other classifiers tested such as VGG16, VGG19, ResNet18. In comparison with earlier MobileNet versions, the 1 × 1 expansion layer taken from the inverted residual unit from the MobileNetV2 is moved beyond the pooling layer, improving upon computation and latency. In addition, MobileNetV3 introduces the swish non-linearity as a replacement to the traditional Rectified Linear Unit (ReLU) as per (1) and (2). Due to the high computational cost of the sigmoid function, a hard swish (h-swish) is introduced in the model as represented by (3). H-swish is more memory and computationally efficient than swish.

1 $$\:sigmoid\left(x\right)=\frac{1}{1+{e}^{-x}}$$

2 $$\:swish\left(x\right)=x.sigmoid\left(x\right)$$

3 $$\:{h}_{swish\left(x\right)}=x\times\:\frac{ReLU(x+3)}{6}$$

### Data pre-processing

**Fig. 3:**
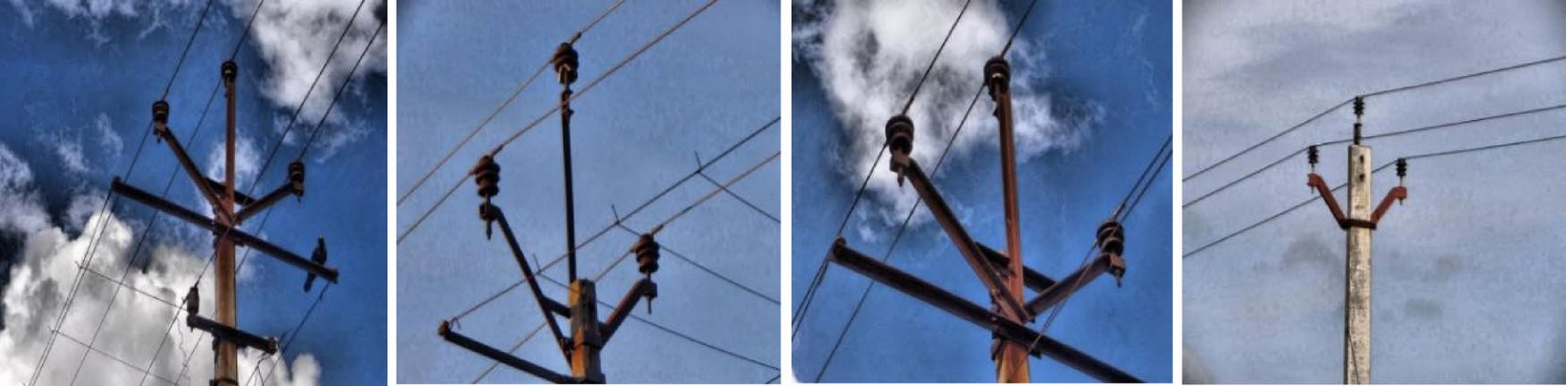
Adaptive equalization technique to enhance contrast. *Step 1: Let an image I be split into non-overlapping boxes of m×n pixels. Step 2: H(i) is the histogram for each block where i represent pixel intensity. Step 3: C(i) is the cumulative distribution function of* $H(i) = \sum_{j=0}^i H(i)$. *Step 4: L(i) is the histogram equalization calculated as L(i) = 1 - Max possible intensity×C(i)*.

To improve on the robustness of the proposed classifier, various pre-processing and data augmentation steps are carried out. These include resizing images to 640 × 640 pixels to improve image quality. Further pre-processing is done by converting images to greyscale. This highlights the contrast of any insulator against its neighbouring surroundings while still highlighting important features. A higher level of colour contrast also enhances data robustness and acts as additional augmentation; hence the adaptive equalization technique is used to enhance contrast as shown in Fig. 3. In this adaptive equalization technique, every image is split into a grid of segmented boxes of an arbitrary pixel size. After this step, the algorithm calculates a cumulative distribution function for the histograms of each pixel intensity. This function is normalized with respect to the maximum possible intensity associated with that pixel set as explained in the following steps. Standard augmentation such as horizontal and vertical flipping is applied along with image rotation and adding noise and blur to the images to simulate real life image imperfections.

### Flowchart

**Fig. 4:**
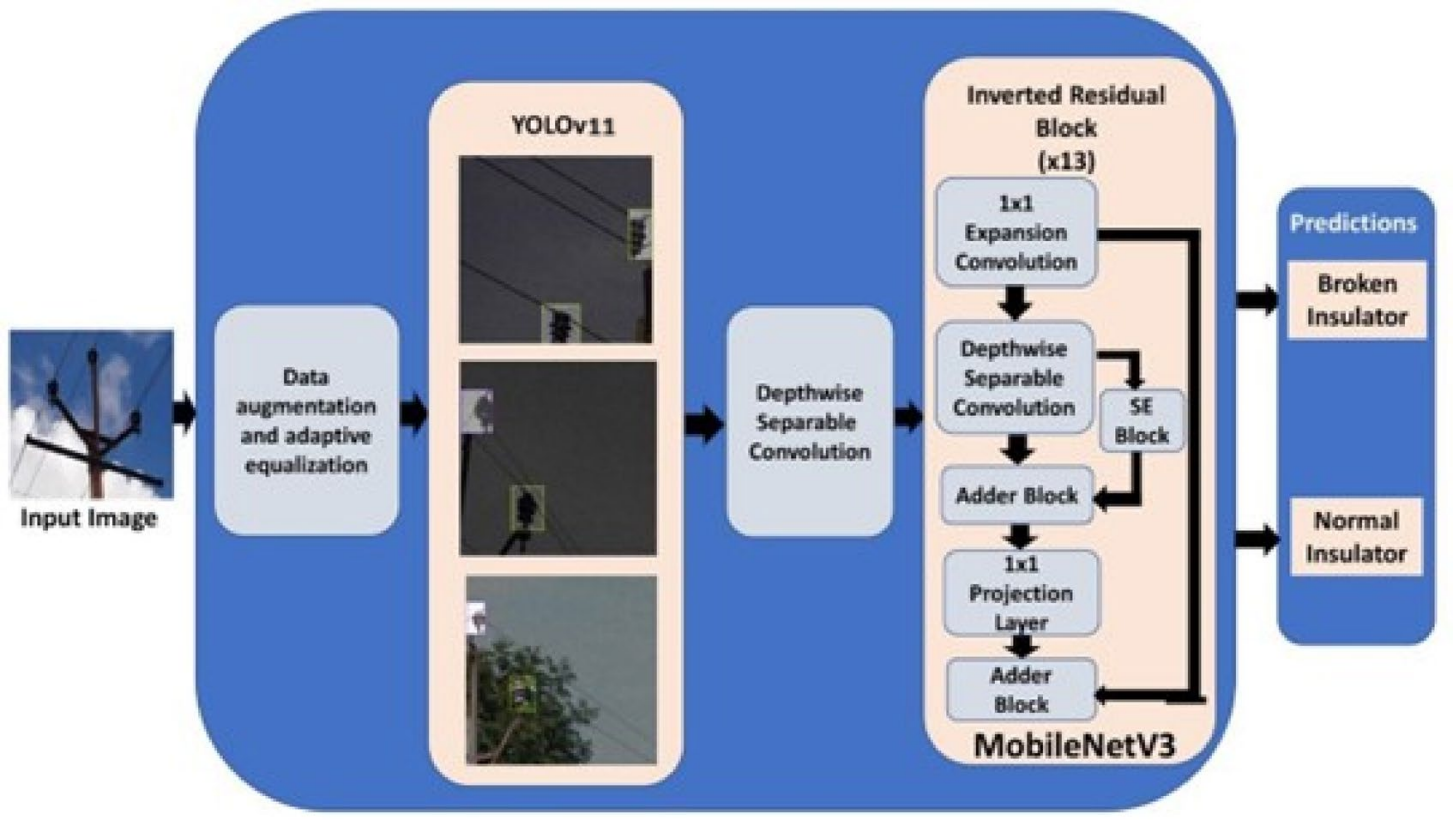
Flowchart of the proposed methodology.

The flowchart of the algorithm is shown in Fig. 4. The proposed model uses a two-step approach. The YOLOv11 model is trained in offline mode with real images as well as the augmented dataset. Similarly, the MobileNetV3 model is also trained offline. After the required training, the trained YOLOv11 is used for online object detection, where it detects the presence of an insulator in the input image, while the trained MobileNetV3 model is used for the online classification of the identified insulator based on any potential breakage. All the input images are pre-processed to make it compatible with the proposed model.

### Training

The dataset contains 1000 images out of which 423 cases are of insulators. The proposed model is trained for 150 epochs. The training of the model is analysed in terms of precision, recall, F1 score and mAP. The corresponding mathematical representations for each are shown in (4) to (6), where True Positive is the probability of positive predictions on positive samples, False Positive is the probability of positive predictions on negative samples (Type 1 error) and False Negative is the probability of negative predictions on positive samples (Type 2 error). mAP is a performance metric, used for object detection methods, which compares the absolute truth of the bounding box to the predicted box and returns a score. mAP can be represented as in (7), where is the number of classes and is the average precision for class *i*. The proposed model has a precision of 0.9903, recall of 0.9919, F1 score of 0.9911 and mAP of 0.9944. This performance can be attributed to the augmented dataset’s enhanced feature extracting abilities by the introduction of additional convolution and max pool layers. The model was trained using an Adam optimizer with an initial learning rate of 0.001. Training was carried out on a standard NVIDIA RTX 3060 GPU.

The testing of the proposed algorithm achieved an inference time and speeds of about 13 FPS on the YOLO model and 30 FPS for MobileNet. These results show feasibility in real-time DAS systems.

**Fig. 5:**
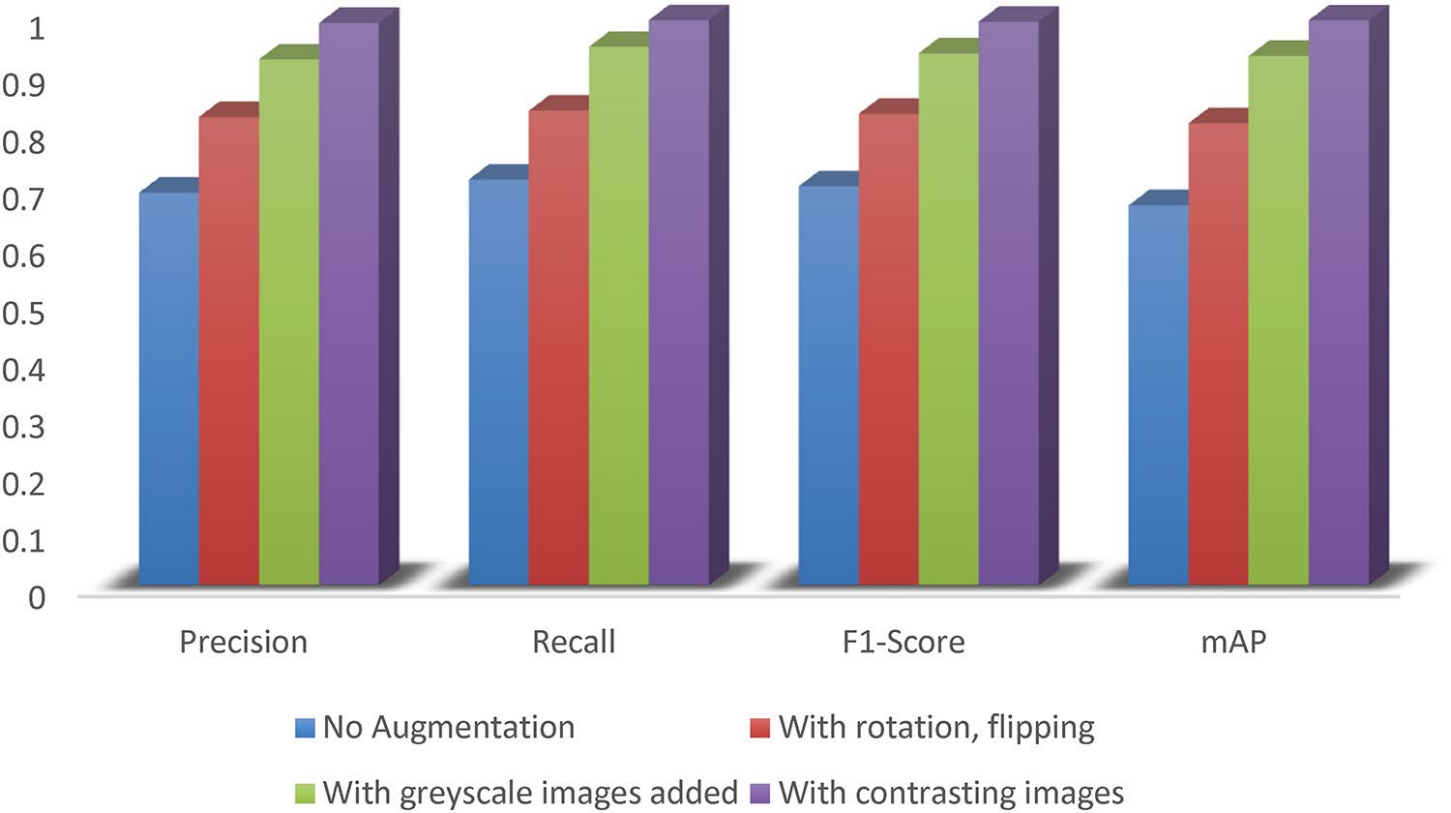
Effect of augmentation in the proposed method.

**Fig. 6:**
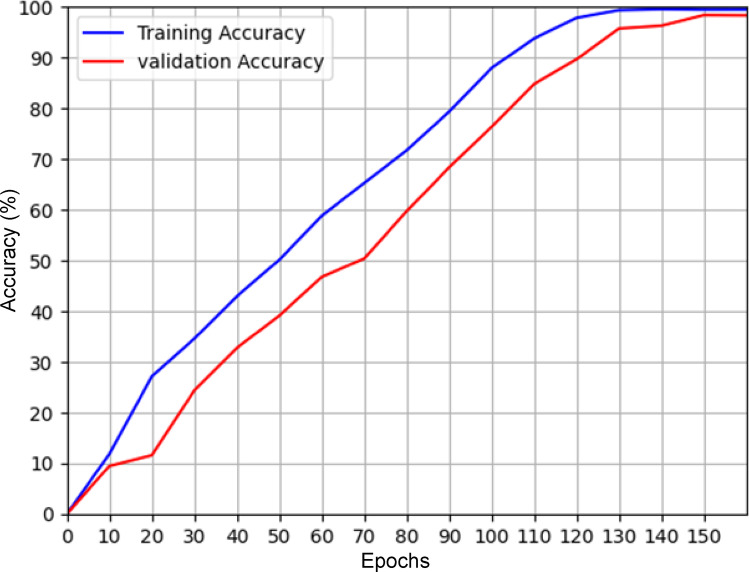
Box Training and validation accuracy.

**Fig. 7:**
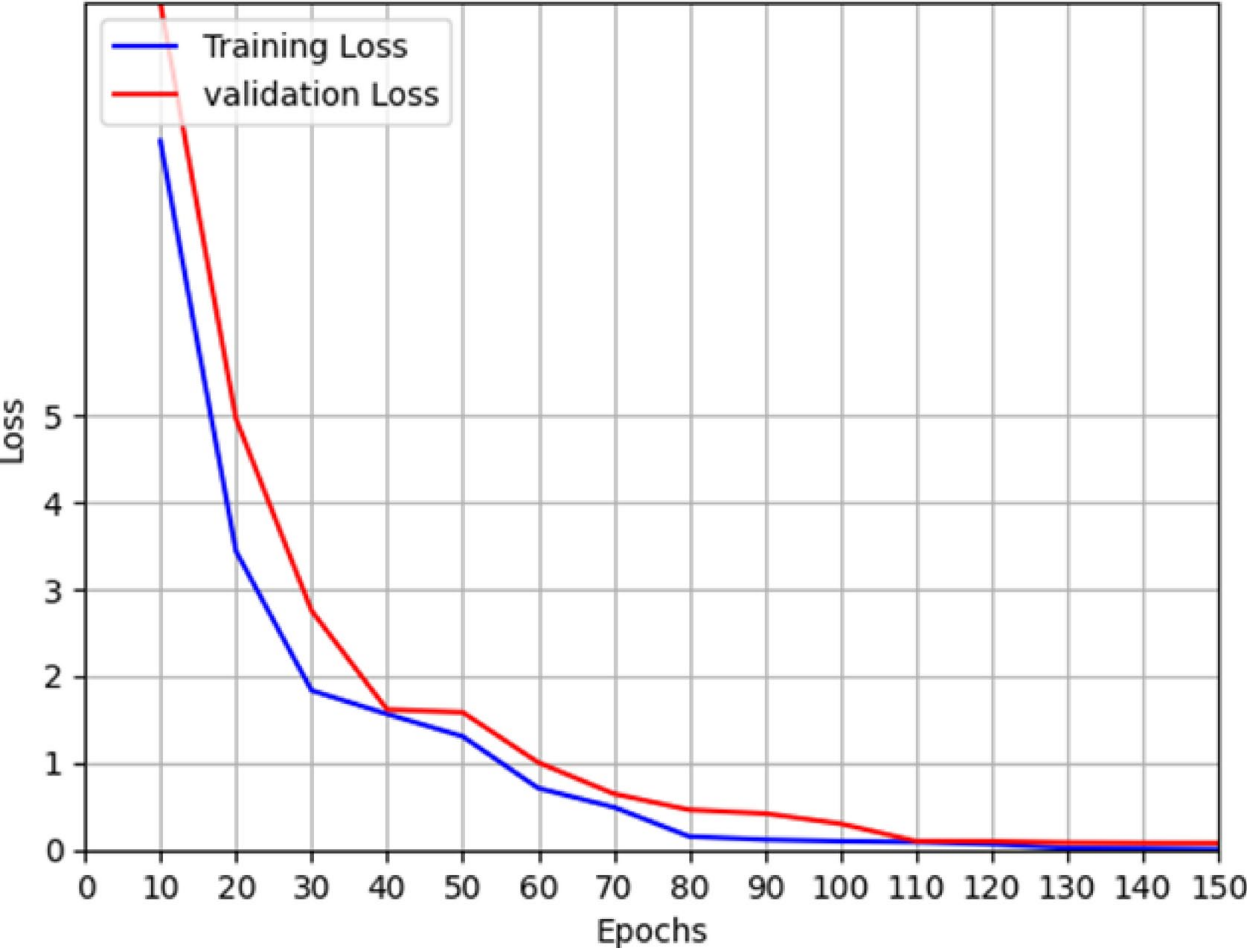
Box Training and validation loss.

**Fig. 8:**
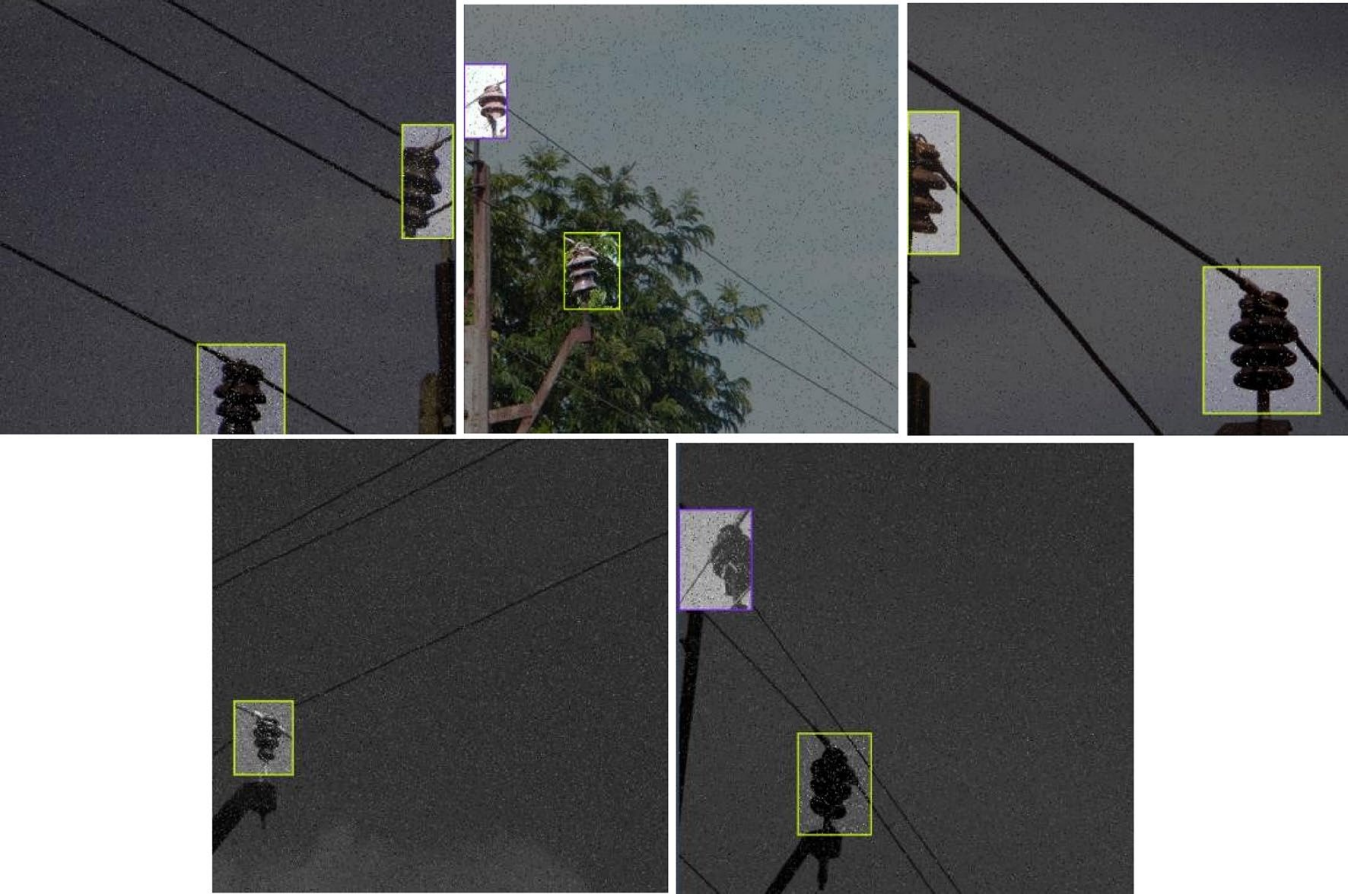
Confusion Matrix for the out of set data samples.

Table 1 shows the comparative results between the proposed model, YOLOv11, YOLOv5 and YOLOv4. It can be seen from the table that the proposed model significantly outperforms YOLOv11, YOLOv5 and YOLOv4 object detection models for the same dataset. The model also remains lightweight as a classifier and object detection algorithm. However, the proposed model has some limitations in terms of training time when compared with YOLOv11. Since, the training is done in offline mode, the training duration is not a major factor while online monitoring of the images. To show the effect of augmentation on the proposed model, the performance of the model for different amounts of augmentation is shown in Fig. 5. The loss and accuracy graphs for the proposed model training are shown in Figs. 6 and 7 respectively. It can be seen that the performance of the proposed method improves significantly with the introduction of the augmented data. The output of YOLOv11 with different degrees of augmentation is shown in Fig. 8. It can be seen that it is able to segregate the insulators from the rest of the objects in the image. The model is further evaluated against other methods over the same dataset in Table 2 which depicts the supremacy of the proposed method over other methods.

4 $$\:Precision=\frac{True\:Positive}{True\:Positive+False\:Positive}$$

5 $$\:Recall=\frac{True\:Positive}{True\:Positive+False\:Negative}$$

6 $$\:F1\:Score=\frac{Precision\times\:Recall\times\:2}{Precision+Recall}$$

7 $$\:mAP=\frac{1}{{n}_{class}}\sum\:_{i=1}^{i={n}_{class}}{AP}_{i}$$

**Table 1:** Comparison of modified YOLOv11 with its other versions.

Model	Batch Size	Precision	Recall	F1 Score	mAP	Training Time (h)
YOLOv4	4	0.9636	0.9712	0.9674	0.9711	12.31
	8	0.9698	0.9677	0.9687	0.9741	9.61
	16	0.9574	0.9523	0.9548	0.9566	13.14
YOLOv5 m	4	0.9655	0.9721	0.9687	0.9765	19.31
	8	0.9677	0.9733	0.9705	0.9788	15.11
	16	0.971	0.9743	0.9726	0.9789	9.75
YOLOv11	4	0.9734	0.9767	0.9751	0.9833	16.88
	8	0.9801	0.9851	0.9825	0.9873	14.9
	16	0.9765	0.9811	0.9787	0.9835	9.45
**Modified YOLOv11**	**4**	**0.9843**	**0.9906**	**0.9874**	**0.9913**	**17.32**
	**8**	**0.9868**	**0.9918**	**0.9893**	**0.9916**	**15.55**
	**16**	**0.9903**	**0.9919**	**0.9911**	**0.9944**	**11.16**

**Table 2:** Different classifier models against the proposed algorithm.

Models	Precision	Recall	F1-Score	mAP
ResNet-50	0.9804	0.9812	0.9808	0.9834
VGG-16	0.9841	0.9845	0.9844	0.9851
DenseNet-121	0.9819	0.9812	0.9815	0.9845
**Modified YOLOv11 model**	**0.9903**	**0.9919**	**0.9911**	**0.9944**

## Results and discussion

### Performance

**Fig. 9:**
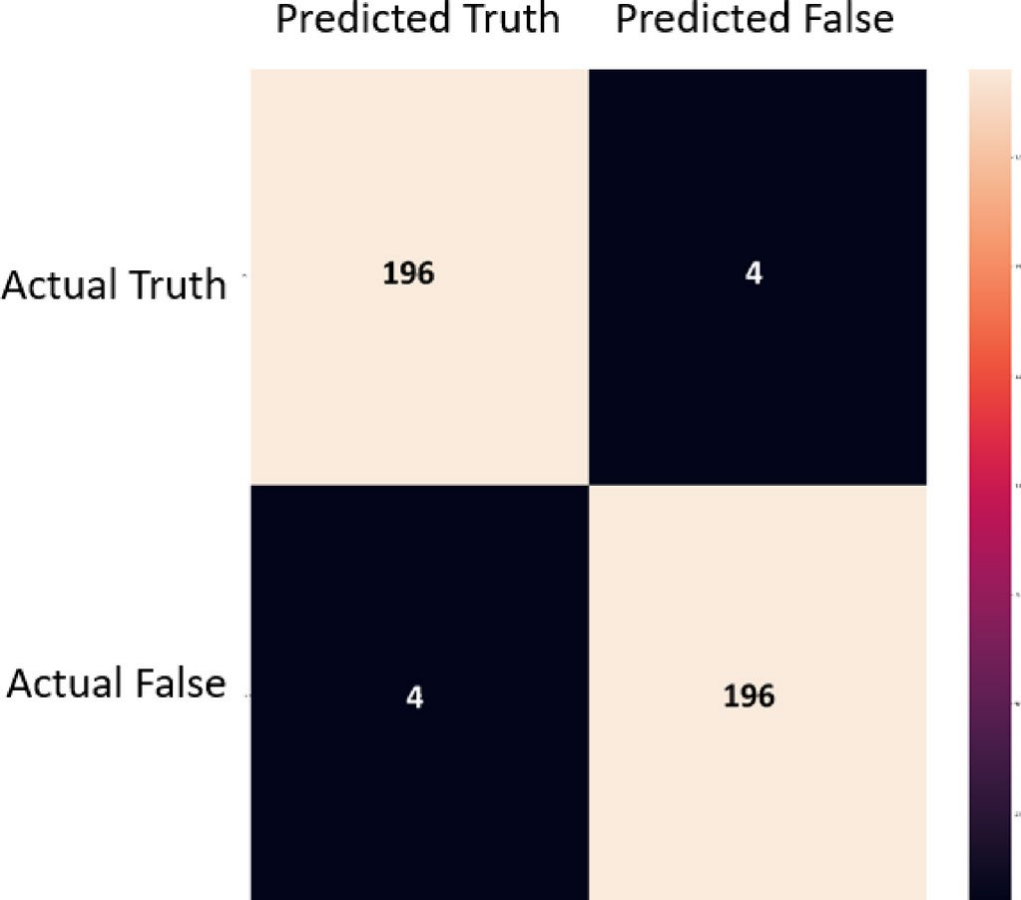
Confusion Matrix for the out of set data samples.

**Fig. 10:**
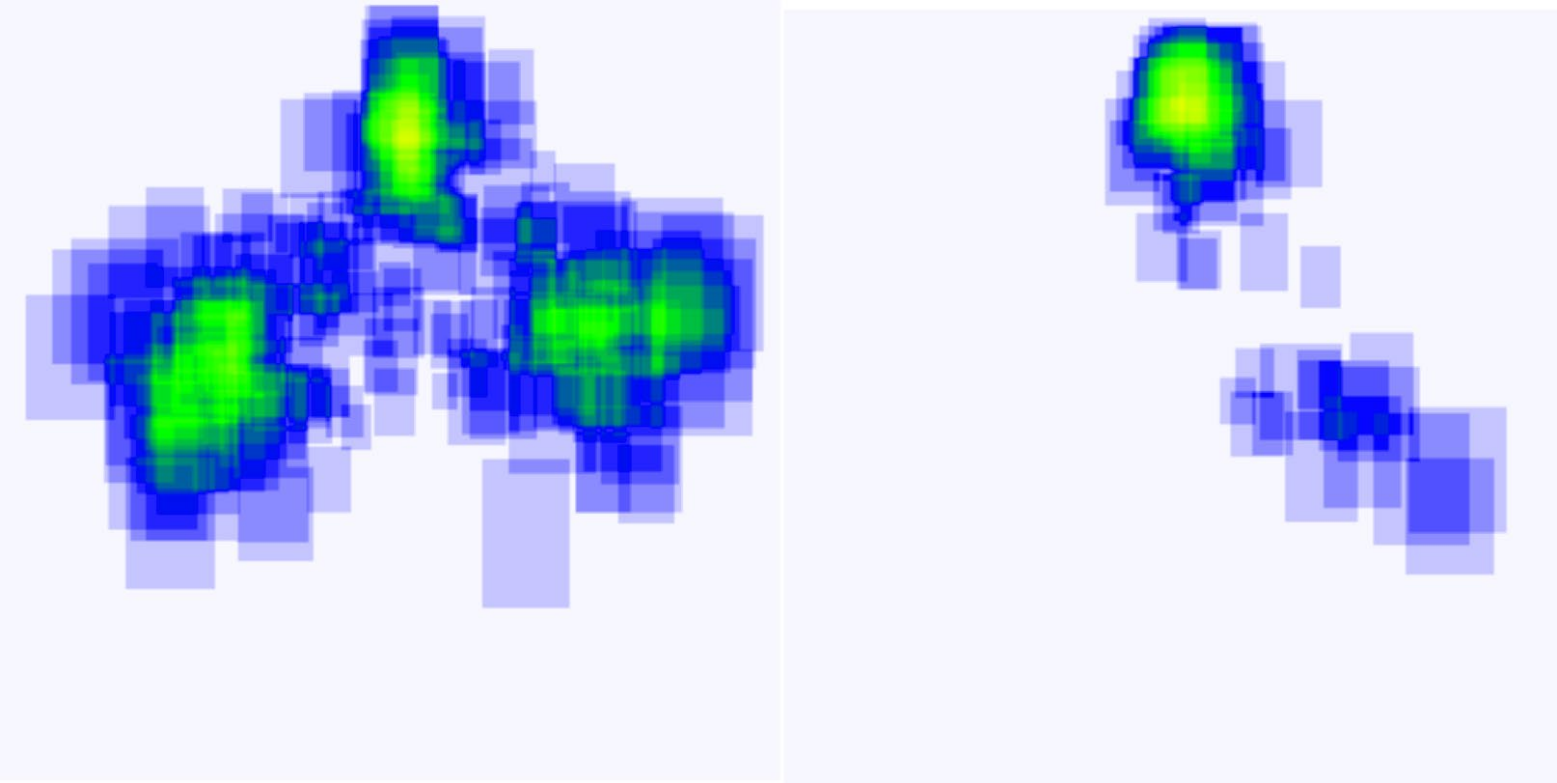
Heatmap to show the efficacy of the proposed method.

**Fig. 11:**
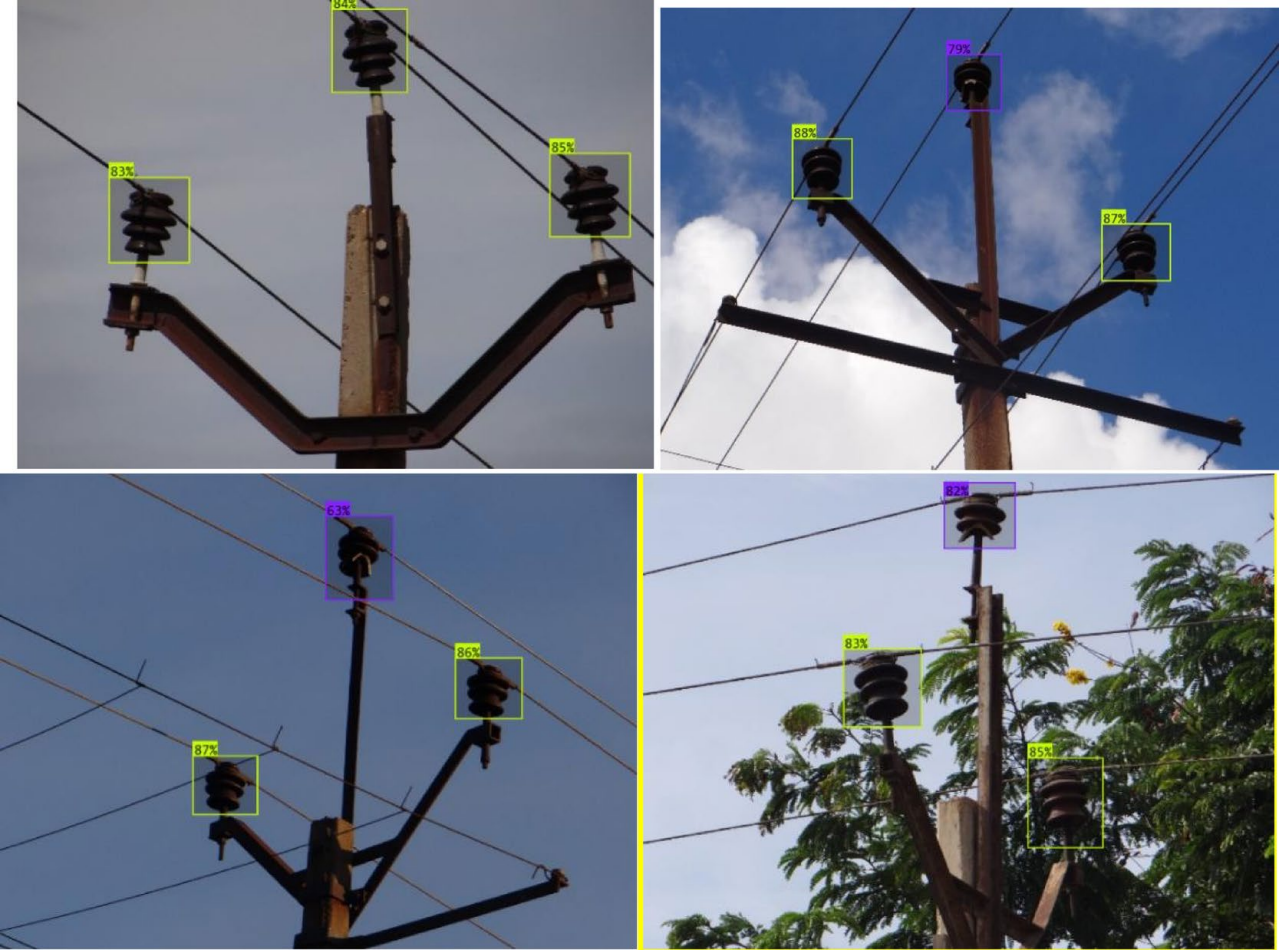
Sample of classification of insulators by the proposed method

To test the developed methodology against out of sample dataset, the proposed methodology was tested against a fresh batch of 400 images. It gives a high accuracy of upto 98%. The confusion matrix for the dataset is given in Fig. 9. It is seen that the false positive rate and type 2 error were both at 2% which demonstrated high precision. For further clarity, the heatmaps are developed in Fig. 10. For this, 5 results were compiled and superimposed. Figure 10(a) indicates the position of insulators as detected by modified YOLOv11 stage while Fig. 10(b) indicates a heatmap of broken insulators as classified by MobileNetV3 model. As seen in Fig. 10(b), broken insulators are seen at a great intensity in the topmost insulator, this corresponds with ground truth observations. Evidently, it correctly identifies three insulators on a pole as well as the defective insulator. A sample classification on these images is shown in Fig. 11. The confidence levels are marked on each insulator which shows that the proposed method exhibits a high confidence level.

### Case studies of complex background conditions

The task of object identification becomes tough when the background has complex conditions. To further test the efficacy of the proposed algorithm for classification of the insulators under various complex background conditions, the following case studies have been considered for testing.

i. Trees: 17 cases where the insulators are in front of trees.
ii. Building: 15 cases where the insulators are in front of buildings.
iii. Whole leaves: 13 cases where the insulators are in leaves of trees.
iv. Water tank: 14 cases where the insulators are in front of water tanks.
v. Yellow leaves: 21 cases where the insulators are in front of trees with yellow leaves, particularly in autumn.
vi. Foggy: 18 cases where the insulators are covered in fog, particularly in winter.
vii. Sun glare: 23 cases where the insulators face the glare of the sun, particularly during the summers.

**Fig. 12:**
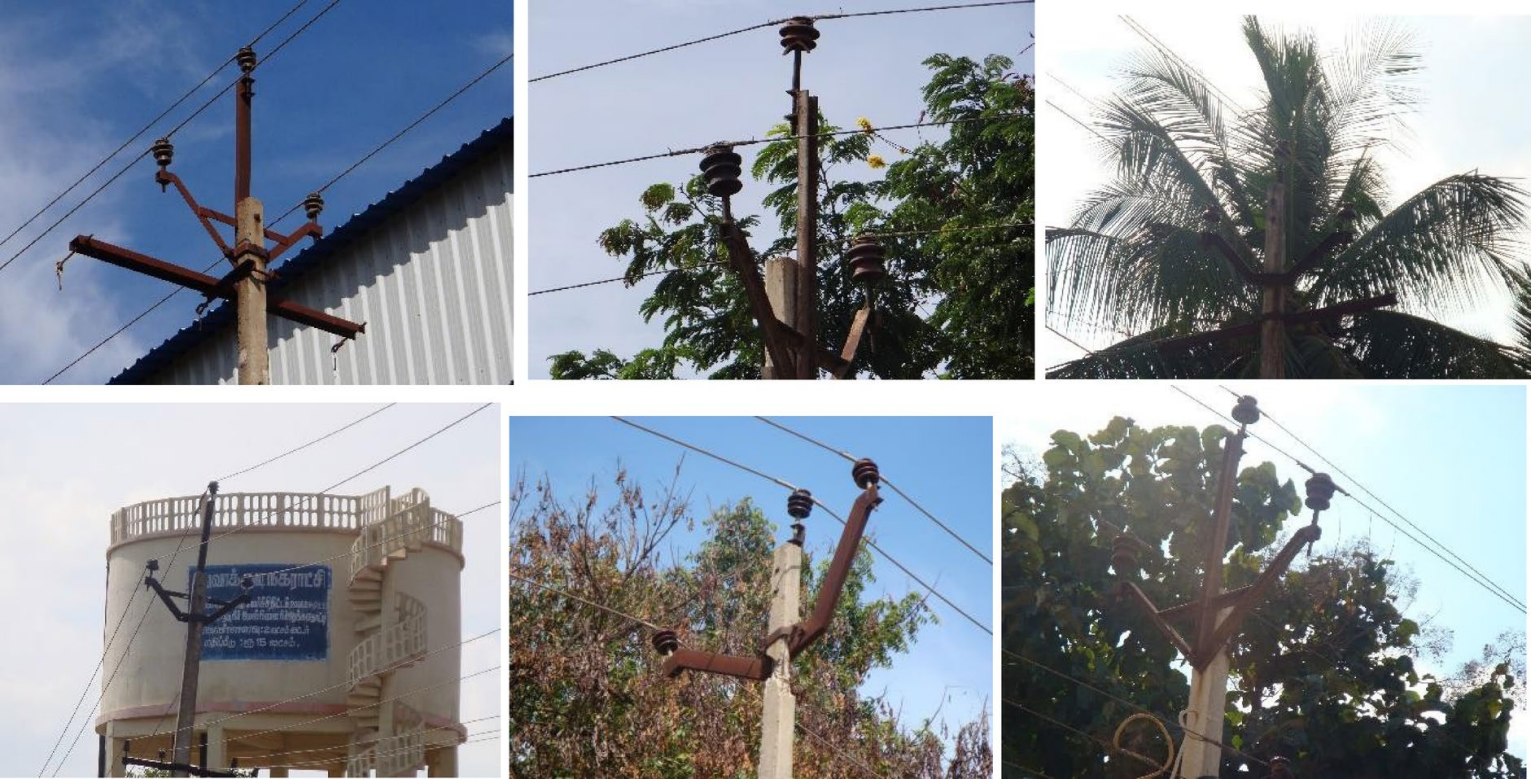
Sample images for different case studies.

**Fig. 13:**
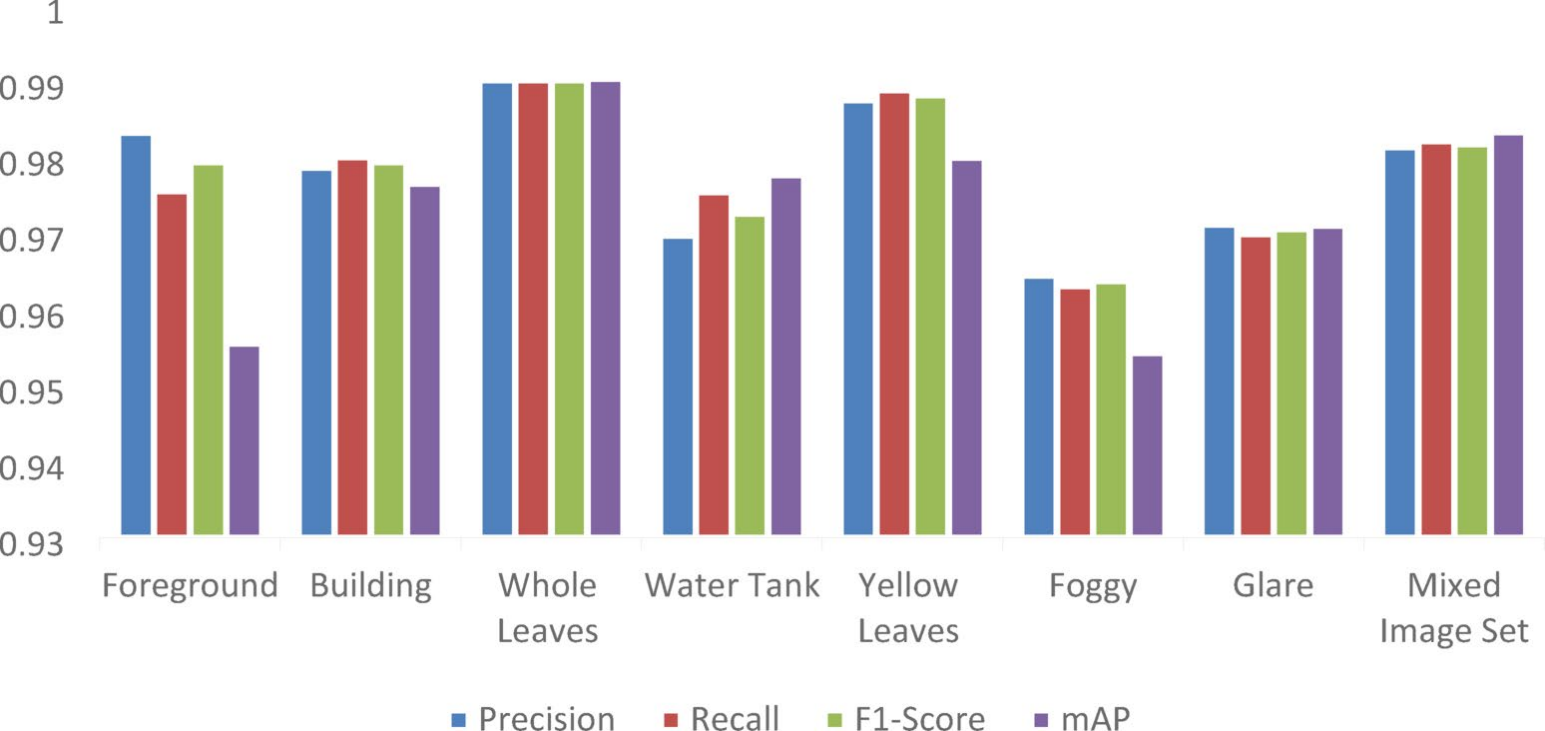
Performance of the proposed method for different case studies.

Different numbers of cases are taken under each category as per the availability of the dataset − 17 case studies for foreground, 15 case studies for building, 13 case studies for whole leaves, 14 case studies for water tank, 21 case studies for yellow leaves, 18 case studies for foggy weather, 23 case studies for glare and 100 case studies for the mixed image set. The sample images for the test cases are shown in Fig. 12. The results of the algorithm are produced in Fig. 13. It can be seen that the proposed algorithm is able to correctly distinguish the faulty insulators even under complex backgrounds. The precision, recall, F1 score and mAP for the mixed dataset is thus found to be 0.9814, 0.9822, 0.9818 and 0.9834 respectively which supports the advantage of the proposed method in terms of ability of implementation all throughout the year irrespective of type of climate or seasons.

### Comparison with other methods

The method is finally compared with some of the methods mentioned in the literature survey as in Table 3. The methods are compared on the basis of metrices which are generally employed to assess the performance of an object detection methods and machine learned methods. These metrices include precision, recall, average precision and mean average precision. All the data are taken from the original literature paper available. It can be seen from the table that the proposed method performs better than the other methods for all the metrices. This performance can be attributed to the dataset’s enhanced feature extracting abilities by the introduction of additional convolution and max pool layers in modified YOLOv11 model and due to the employment of MobileNetV3 as an ideal lightweight classifier for classification.

**Table 3:** Comparison of other deep learning methods with proposed method.

Model	Year	Dataset	Evaluation Parameters (%)					
			Precision	Recall	AP	AP50	AP75	Map
Box-point^6^	2021	Private	-	-	73.7	94	86.4	-
MTI-YOLO^24^	2021	CCIN	95	91	89.72	-	-	-
YOLOv3^22^	2022	CPLID	90	91	-	-	-	-
Improved^22^ YOLOv3^22^	2022	CPLID	94	92	-	-	-	-
YOLOv4^23^	2022	CPLID	-	-	93.84	-	-	94.39
YOLOv5^23^	2022	CPLID	-	-	95.67	-	-	95.31
RIIR-Net^28^	2022	Private	74	75	-	-	-	98
YOLOD^26^	2023	IDID	-	-	73.9	98	84.6	-
**Proposed**	**2025**	**Private**	**99.03**	**99.19**	**99.54**	**99.87**	**99.41**	**99.44**

Thus, the core characteristics of the developed model can be summarized as follows:

1. The authors utilize the three highly specialized blocks introduced in the YOLOv11 model, namely the C3 K2, SPFF and C2PSA blocks. These blocks improve the detection of small objects such as broken insulators while keeping the model resilient to background noise.
2. The authors integrate a MobileNetV3 block classifier into the proposed algorithmic pipeline as the primary classifier to identify a detected insulator as either faulty or healthy, having it optimized for deployment.
3. A highly varied and diverse dataset of 11 kV insulators collected using UAVs at NIT Trichy’s campus, including various insulator conditions involving glare, fog, noise and blur, have been employed.
4. The proposed algorithm has been benchmarked against previous YOLO versions as well as YOLOv11, showing remarkable improvement.

## Conclusion

The modern power system is in a new era of healthy, sustainable and fast development, specifically in the distribution side. With the paradigm shift into active distribution networks, the health of the distribution insulators is becoming increasingly important for power reliability. These insulators are constantly exposed to climatic variations and aging which leads to their health deterioration in the long term. After a certain level, the poor health of the insulators becomes problematic for a reliable power flow and needs to be replaced. This paper deals with an automatic detection of insulators defect with deep neural network. For this, YOLOv11 is used as the deep learning model along with MobileNetV3. The dataset is developed from the images available from an 11 kV distribution line. To increase the number of samples required to properly train YOLOv11, data augmentation is implemented. Several case studies have been implemented to show the strength of the algorithm. The proposed algorithm has also been compared with other deep learned models and earlier version of YOLO for insulator fault detection. The evaluation metrics show the supremacy of the proposed method over other methods. Though the authors proposed the idea of implementing DAS for insulator fault detection, the practical applicability derived from inference on mobile devices or servers can be taken as the future work. From an engineering perspective, using a more powerful terminal server for unified inference would enable the implementation of advanced detection algorithms, a capability that the DAS architecture is specifically designed to support. Meta learning and hybrid knowledge of insulator defect detection algorithms can also be regarded as the future extended work of the presented algorithm.

## Data Availability

The data that support the findings of this study are available from the corresponding author upon reasonable request.
